# Socio-affective skills and early academic disengagement in higher education: a sequential mediation model of emotion regulation and academic self-efficacy

**DOI:** 10.3389/fpsyg.2026.1849945

**Published:** 2026-07-23

**Authors:** Karen Jimenez Arriaga, David Aaron Miranda Garcia, Diego Yael Gonzalez Gomez

**Affiliations:** 1 School of Health Sciences, Universidad del Valle de México, Mexico City, Mexico; 2 Center for Multidisciplinary Research in Education, Universidad Autónoma del Estado de México, Toluca, Mexico

**Keywords:** academic self-efficacy, early academic disengagement risk, emotion regulation difficulties, psychological disengagement, socio-affective skills

## Abstract

Student disengagement in higher education is increasingly conceptualized as a gradual, psychologically mediated process that affects academic functioning and persistence, rather than a discrete academic decision. Within this framework, early academic disengagement (previously conceptualized as silent dropout processes) refers to early psychological disengagement that precedes formal withdrawal. This study examined the psychological pathways linking socio-affective skills to early academic disengagement risk through emotion regulation and academic self-efficacy. A cross-sectional design was employed with a sample of 942 first-year undergraduate students enrolled at a private university in Mexico. Participants completed standardized measures of socio-affective skills, emotion regulation difficulties (DERS), academic self-efficacy, and early academic disengagement risk. Structural equation modeling was used to examine a theory-informed sequential mediation framework. Results indicated that socio-affective skills were indirectly associated with lower early academic disengagement risk through reduced emotion regulation difficulties and higher academic self-efficacy, with a significant total indirect effect (−0.32, 95% *CI* [−0.38, −0.26]) and a significant sequential indirect effect (−0.05, 95% *CI* [−0.08, −0.03]). The final model demonstrated good fit (*CFI* = 0.95, *RMSEA* = 0.038) and explained 47% of the variance in early academic disengagement risk, with the direct effect reduced but remaining significant, indicating partial mediation. These findings suggest that emotional and motivational variables are meaningfully associated with early academic disengagement risk. These findings may inform future efforts aimed at early identification and intervention strategies in higher education.

## Introduction

University dropout is increasingly understood as a complex, gradual, and psychologically mediated process rather than a discrete academic decision. Accordingly, contemporary research has shifted its focus toward early forms of disengagement that precede formal withdrawal and often remain invisible to institutional monitoring systems. Within this perspective, the concept of *early academic disengagement (silent dropout processes)* has gained relevance as a way of describing a pre-decisional phase marked by emotional exhaustion, reduced academic effort, avoidance of evaluative situations, and a progressive weakening of academic identification, despite continued enrollment (Rump et al., 2023; Álvarez-Pérez et al., 2024).

Importantly, growing empirical evidence suggests that academic disengagement risk may represent an important vulnerability marker within students' educational experiences. Studies have shown that early psychological disengagement is associated with dropout intentions, lower academic performance, and poorer psychological well-being (Kahu and Nelson, 2018; Pedler et al., 2022; Gilani and Thomas, 2025). Consequently, contemporary research has increasingly focused on identifying psychological processes associated with disengagement vulnerability, particularly during the first year of higher education.

From this standpoint, individual psychological resources that enable students to cope adaptively with academic and social demands have become central to contemporary dropout research. Among these resources, socio-affective skills have emerged as particularly salient within social–emotional learning and self-regulation frameworks. These skills encompass emotional awareness, empathy, assertive communication, cooperation, and adaptive interpersonal functioning. Recent evidence suggests that such competencies are systematically associated with higher levels of academic engagement, persistence, and adjustment in higher education contexts (Al-Maamari et al., 2025; Taylor et al., 2017).

Nevertheless, current theoretical accounts caution against conceptualizing socio-affective skills as direct determinants of dropout-related outcomes. Rather, these skills are more appropriately understood as distal protective resources whose influence unfolds through more proximal psychological mechanisms that govern students' daily academic functioning (Putwain et al., 2022). In this regard, emotion regulation and academic self-efficacy have been consistently identified as central mechanisms of academic persistence, yet they have rarely been examined jointly within a unified explanatory model of early academic disengagement risk.

Emotion regulation occupies a pivotal position within this proposed pathway. Defined as the processes through which individuals monitor, evaluate, and modify emotional responses to meet situational demands, emotion regulation is particularly critical in academic environments characterized by frequent evaluation, uncertainty, and social comparison (Gross, 2015). Recent studies demonstrate that emotion regulation difficulties are strongly associated with disengagement behaviors, avoidance coping strategies, and other maladaptive academic behaviors, which have been linked to later dropout intentions in longitudinal research (Balkis and Duru, 2022; Gilani and Thomas, 2025).

Crucially, socio-affective skills appear to foster more adaptive emotion regulation by enhancing emotional awareness, perspective-taking, and the effective use of social support. Empirical findings indicate that students with stronger socio-affective competencies report lower levels of emotional dysregulation and academic stress, alongside greater use of adaptive regulation strategies such as cognitive reappraisal (Liu et al., 2022; Zhou and Ee, 2012). These patterns are consistent with the possibility that emotion regulation may be associated with the relationship between socio-affective skills and early academic disengagement risk, although the cross-sectional nature of available evidence does not allow confirmation of temporal sequencing.

At the same time, academic self-efficacy represents a second, closely interconnected psychological pathway. Grounded in social cognitive theory (Bandura, 1997; Zimmerman, 2000), academic self-efficacy refers to students' beliefs in their capacity to successfully organize and execute academic tasks. Recent research consistently identifies low academic self-efficacy as a robust predictor of disengagement, procrastination, and dropout intentions, even after controlling for prior academic achievement (Honicke and Broadbent, 2016, 2023).

Emerging longitudinal evidence further suggests that emotion regulation and academic self-efficacy are dynamically intertwined. Students who regulate academic emotions effectively are more likely to maintain confidence in their academic capabilities following setbacks, whereas persistent emotional dysregulation tends to undermine self-efficacy beliefs and accelerate disengagement trajectories (Liu et al., 2022). Within this process, socio-affective skills play a foundational role by enabling constructive feedback processing, adaptive social comparison, and sustained motivation in evaluative contexts.

Consequently, the present study examines whether the associations among socio-affective skills, emotion regulation difficulties, academic self-efficacy, and early academic disengagement risk are consistent with a theoretically informed sequential mediation framework. By explicitly modeling these psychological pathways, this research addresses a critical gap in the literature and contributes to the development of theoretically grounded frameworks for early identification and prevention of dropout risk in higher education.

Accordingly, the following hypotheses were tested:

*H1: Socio-affective skills are negatively associated with early academic disengagement risk*.

*H2: Emotion regulation difficulties are expected to be significantly associated with the relationship between socio-affective skills and early academic disengagement risk*.

*H3: Academic self-efficacy is expected to be significantly associated with the relationship between socio-affective skills and early academic disengagement risk*.

*H4: The pattern of associations among socio-affective skills, emotion regulation difficulties, academic self-efficacy, and early academic disengagement risk is expected to be consistent with a theoretically informed sequential mediation model*.

## Method

### Design

The present study employed a cross-sectional, correlational design aimed at examining the psychological pathways linking socio-affective skills to early academic disengagement risk through the mediating roles of emotion regulation and academic self-efficacy. This design is consistent with contemporary research examining mediation mechanisms underlying academic disengagement and dropout-related outcomes in higher education (Álvarez-Pérez et al., 2024; Honicke and Broadbent, 2023).

Although the proposed model specifies theoretically informed associations among socio-affective skills, emotion regulation difficulties, academic self-efficacy, and early academic disengagement risk, the cross-sectional nature of the data does not permit conclusions regarding temporal precedence or causal ordering among these variables. Consequently, the model should be interpreted as a theory-informed representation of associations rather than confirmation of a developmental process.

### Participants

A total of 978 first-year undergraduate students initially completed the survey. Following data screening procedures, 36 cases (3.7%) were excluded due to incomplete responses on one or more core study variables, resulting in a final analytic sample of 942 students. Participants ranged in age from 17 to 20 years (*M* = 18.41, *SD* = 0.76). The sample was predominantly female (*n* = 634, 67.3%), whereas 308 participants (32.7%) identified as male. Students were enrolled across five academic fields: Health Sciences (*n* = 320; 34.0%), Engineering (*n* = 207; 22.0%), Tourism and Gastronomy (*n* = 122; 13.0%), Design (*n* = 198; 21.0%), and Arts and Architecture (*n* = 95; 10.1%).

Eligibility criteria included: (a) enrollment in the first academic year of an undergraduate program, (b) full-time student status at the time of data collection, and (c) voluntary agreement to participate in the study. Missing data represented 3.7% of the original dataset. Given the relatively low proportion of missingness (3.7%) and the absence of any observable systematic pattern during data screening, listwise deletion was considered appropriate for subsequent analyses. Consistent with commonly accepted recommendations, listwise deletion is unlikely to introduce substantial bias when the proportion of missing data is below 5%.

### Measures

#### Socio-affective skills (SECQ)

Socio-affective skills were assessed using the Social Emotional Competence Questionnaire (SECQ; Zhou and Ee, 2012), a self-report instrument grounded in social–emotional learning frameworks. The SECQ comprises 25 items covering five dimensions: self-awareness, social awareness, self-management, relationship management, and responsible decision-making. Responses are recorded on a 5-point Likert scale ranging from 1 (strongly disagree) to 5 (strongly agree), with higher scores indicating greater social–emotional competence.

Item administration and scoring. Items were averaged to compute a total SECQ score; dimension scores were computed by aggregating the corresponding items. Negatively worded items were reverse-coded prior to scoring. In the present sample, internal consistency was good (Cronbach's α = 0.86).

The full instrument characteristics are reported: number of items (25), response format (1–5), and scoring rules (reverse coding and aggregation). If required, the complete item list and scoring key are available in the Supplementary materials.

Reliability analyses for individual SECQ dimensions yielded satisfactory internal consistency estimates: self-awareness (ω = 0.84), social awareness (ω = 0.82), self-management (ω = 0.86), relationship management (ω = 0.85), and responsible decision-making (ω = 0.81). Detailed reliability statistics are reported in Supplementary Table S1.

#### Emotion regulation

Emotion regulation difficulties were measured using the Difficulties in Emotion Regulation Scale (DERS) (Gratz and Roemer, 2004). The DERS is a 36-item self-report instrument designed to assess multiple facets of emotional dysregulation, including non-acceptance of emotional responses, difficulties engaging in goal-directed behavior, impulse control difficulties, lack of emotional awareness, limited access to emotion regulation strategies, and lack of emotional clarity. Participants responded using a 5-point Likert scale ranging from 1 (*almost never*) to 5 (*almost always*), with higher scores reflecting greater difficulties in emotion regulation.

The DERS has demonstrated strong psychometric properties across diverse university samples and has been extensively used in studies examining academic stress, disengagement, and related maladaptive academic behaviors (Balkis and Duru, 2022; Liu et al., 2022). In the present study, the total scale score exhibited excellent internal consistency (Cronbach's α = 0.91).

Reliability estimates for the DERS dimensions were also satisfactory: non-acceptance (ω = 0.87), goals (ω = 0.84), impulse (ω = 0.85), awareness (ω = 0.76), strategies (ω = 0.89), and clarity (ω = 0.81). Detailed psychometric information is presented in Supplementary Table S1.

#### Academic self-efficacy

Academic self-efficacy was measured using the Academic Self-Efficacy Scale validated in university populations by Honicke and Broadbent (2016). The scale consists of 6 items assessing students' perceived capability to successfully manage academic demands (e.g., “*I am confident in my ability to meet the academic requirements of my program*”). Items were rated on a 5-point Likert scale (1 = *Strongly disagree* to 5 = *Strongly agree*). Scores were computed as the mean of item responses, with higher scores reflecting stronger academic self-efficacy. In the present sample, internal consistency was high (α = 0.88). Confirmatory factor analysis (CFA) results supported a unidimensional structure, χ^2^ (9) = 18.42, *p* = 0.031, *CFI* = 0.99, *RMSEA* = 0.038, *SRMR* = 0.024.

#### Early academic disengagement risk

Early academic disengagement risk was assessed using an 8-item scale designed to capture early psychological disengagement from academic activities while students remain formally enrolled. Items reflect emotional withdrawal, reduced academic investment, avoidance of evaluative situations, and weakened academic identification (e.g., “*I feel mentally disconnected from my studies even though I am still enrolled”*, “*I often consider disengaging from academic activities without formally dropping out”*). Responses were recorded on a 5-point Likert scale (1 = Strongly disagree to 5 = Strongly agree). A total score was computed as the mean of item responses, with higher scores indicating greater early academic disengagement risk. Item content was generated to reflect process-oriented disengagement characteristics described in prior conceptual and empirical work on early higher-education disengagement (e.g., Rump et al., 2023; Álvarez-Pérez et al., 2024). Items were independently reviewed by two researchers with expertise in higher-education psychology to evaluate content relevance, clarity, and conceptual representativeness. Following expert review, a pilot administration was conducted with a small group of first-year university students to assess comprehensibility and response variability. The complete item wording is provided in Supplementary Appendix A. Scoring procedures are reported in Supplementary Appendix B, and parcel allocation rules are reported in Supplementary Tables S2, S3.

A confirmatory factor analysis (CFA) supported a unidimensional structure, χ^2^ (20) = 46.18, *p* < 0.001, *CFI* = 0.98, *TLI* = 0.97, *RMSEA* = 0.041 (90% *CI* [0.026, 0.056]), *SRMR* = 0.031. Standardized factor loadings ranged from.62 to.81. Composite reliability was adequate (*CR* = 0.88), and average variance extracted indicated acceptable convergent validity (*AVE* = 0.54). Internal consistency in the present sample was high (Cronbach's α = 0.86).

#### Control variables

Age, gender, and academic field were included as control variables in the structural model to account for potential confounding effects on disengagement-related outcomes. Academic field was treated as a categorical covariate and dummy coded prior to analysis, with Health Sciences serving as the reference category. This approach allowed the examination of potential differences across academic disciplines while preserving the categorical nature of the variable.

#### Procedure

Data collection took place during the academic semester through an online questionnaire administered via a secure institutional platform. Participants received a brief explanation of the study objectives and provided informed consent prior to participation. Participation was anonymous, and no personally identifiable information was collected. The average completion time of the questionnaire was approximately 15 min.

#### Ethical considerations

The study was conducted in accordance with the ethical principles outlined in the Declaration of Helsinki and complied with national and institutional guidelines for research involving human participants. The research protocol was reviewed and approved by the Institutional Ethics Committee of the participating university. Participants were informed of the voluntary nature of their participation, their right to withdraw at any time without penalty, and the confidentiality of their responses.

#### Preliminary analyses

To examine potential common method bias, we compared the hypothesized measurement model with a single-factor model. The single-factor model showed substantially poorer fit, suggesting that common method variance is unlikely to fully account for the observed associations.

Prior to model estimation, data were screened for missing values, univariate normality, and outliers. Participants with incomplete responses on the core study variables were excluded from the analyses using listwise deletion, as specified in the Participants section. Examination of skewness and kurtosis values indicated that all observed variables were within acceptable ranges (|skewness| < 2; |kurtosis| < 7), supporting the use of maximum likelihood estimation, which is the default estimation method in AMOS.

Descriptive statistics and bivariate Pearson correlations were computed to examine initial associations among the study variables and to inform model specification.

### Data analysis

SEM analyses were conducted in IBM SPSS AMOS version 29.0 to test the hypothesized direct and indirect relationships among socio-affective skills, emotion regulation difficulties, academic self-efficacy, and early academic disengagement risk.

#### Measurement quality

For descriptive analyses, scale scores were computed as the mean of item responses. For SEM, constructs were specified as **latent variables** using subscale indicators (SECQ, DERS) and item parcels (academic self-efficacy, early academic disengagement risk). Reliability was evaluated with Cronbach's alpha, and measurement model fit and standardized loadings were inspected to support construct validity prior to testing the structural paths.

#### Structural model specification

The hypothesized sequential mediation model was specified in AMOS with socio-affective skills entered as the exogenous variable, emotion regulation difficulties and academic self-efficacy specified as sequential mediators, and early academic disengagement risk modeled as the endogenous outcome variable. Direct paths were estimated from socio-affective skills to emotion regulation difficulties, academic self-efficacy, and early academic disengagement risk; from emotion regulation difficulties to academic self-efficacy and early academic disengagement risk; and from academic self-efficacy to early academic disengagement risk.

All study constructs were modeled as latent variables. Parceling and indicators were specified as follows: For SECQ, the five subscale scores (self-awareness, social awareness, self-management, relationship management, responsible decision-making) were used as indicators of the latent socio-affective skills factor. For DERS, the six subscale scores (non-acceptance, goals, impulse, awareness, strategies, clarity) were used as indicators of the latent emotion regulation difficulties factor. For academic self-efficacy and early academic disengagement risk, three item parcels were created using the item-to-construct balance approach (Little et al., 2002). This procedure distributes item content and factor loadings evenly across parcels, improving indicator reliability, reducing random measurement error, and facilitating stable estimation of latent constructs within structural equation models. Parcel construction rules and item–parcel allocation procedures are reported in Supplementary Tables S2, S3.

The measurement model demonstrated good fit to the data, χ^2^ (224) = 512.67, *p* < 0.001, *CFI* = 0.96, *TLI* = 0.95, *RMSEA* = 0.042 (90% *CI* [0.037, 0.046]), *SRMR* = 0.039. All standardized factor loadings were significant (*p* < 0.001) and exceeded 0.60. Discriminant validity was supported, with all inter-construct correlations below 0.75 and AVE values exceeding squared inter-factor correlations.

#### Indirect effects

Indirect effects were examined using bias-corrected bootstrapping procedures with 5,000 resamples, as implemented in AMOS. Standardized indirect effects and 95% confidence intervals were computed for: (a) the indirect effect of socio-affective skills on early academic disengagement risk via emotion regulation difficulties, (b) the indirect effect via academic self-efficacy, and (c) the sequential indirect effect via emotion regulation difficulties and academic self-efficacy. Indirect effects were considered statistically significant when the bootstrapped confidence interval did not include zero.

#### Model fit evaluation

Model fit was evaluated using multiple fit indices following current recommendations for structural equation modeling (Kline, 2016) reporting standards: the chi-square statistic (χ^2^), the Comparative Fit Index (CFI), the Tucker–Lewis Index (TLI), the Root Mean Square Error of Approximation (RMSEA) with 90% confidence intervals, and the Standardized Root Mean Square Residual (SRMR). Following conventional guidelines, CFI and TLI values ≥0.95, RMSEA values ≤ 0.06, and SRMR values ≤ 0.08 were interpreted as indicating good model fit, while considering overall model complexity and sample size.

#### Control variables

Age, gender, and academic field were included as covariates predicting mediators and the outcome. Academic field was represented by four dummy-coded variables, with Health Sciences serving as the reference category. The inclusion of covariates did not meaningfully alter the magnitude or statistical significance of the primary structural paths. Gender showed a small association with emotion regulation difficulties (β = 0.07, *p* = 0.042), whereas age and all academic field dummy variables were not significantly associated with early academic disengagement risk (all *ps* > 0.10).

## Results

Descriptive statistics and bivariate correlations among the study variables are presented in Table 1.

**Table 1:** Descriptive statistics and bivariate correlations among study variables (*N* = 942).

Variable	*M*	*SD*	1	2	3	4
1. Socio-affective skills	3.74	0.56	–			
2. Emotion regulation difficulties	2.89	0.61	−0.42^***^	–		
3. Academic self-efficacy	3.68	0.59	0.51^***^	−0.46^***^	–	
4. Early academic disengagement risk	2.47	0.64	−0.39^***^	0.58^***^	−0.54^***^	–

Pearson correlation coefficients are reported. Higher scores on emotion regulation indicate greater difficulties.

^***^p < 0.001.

### Structural equation modeling

**Figure 1:**
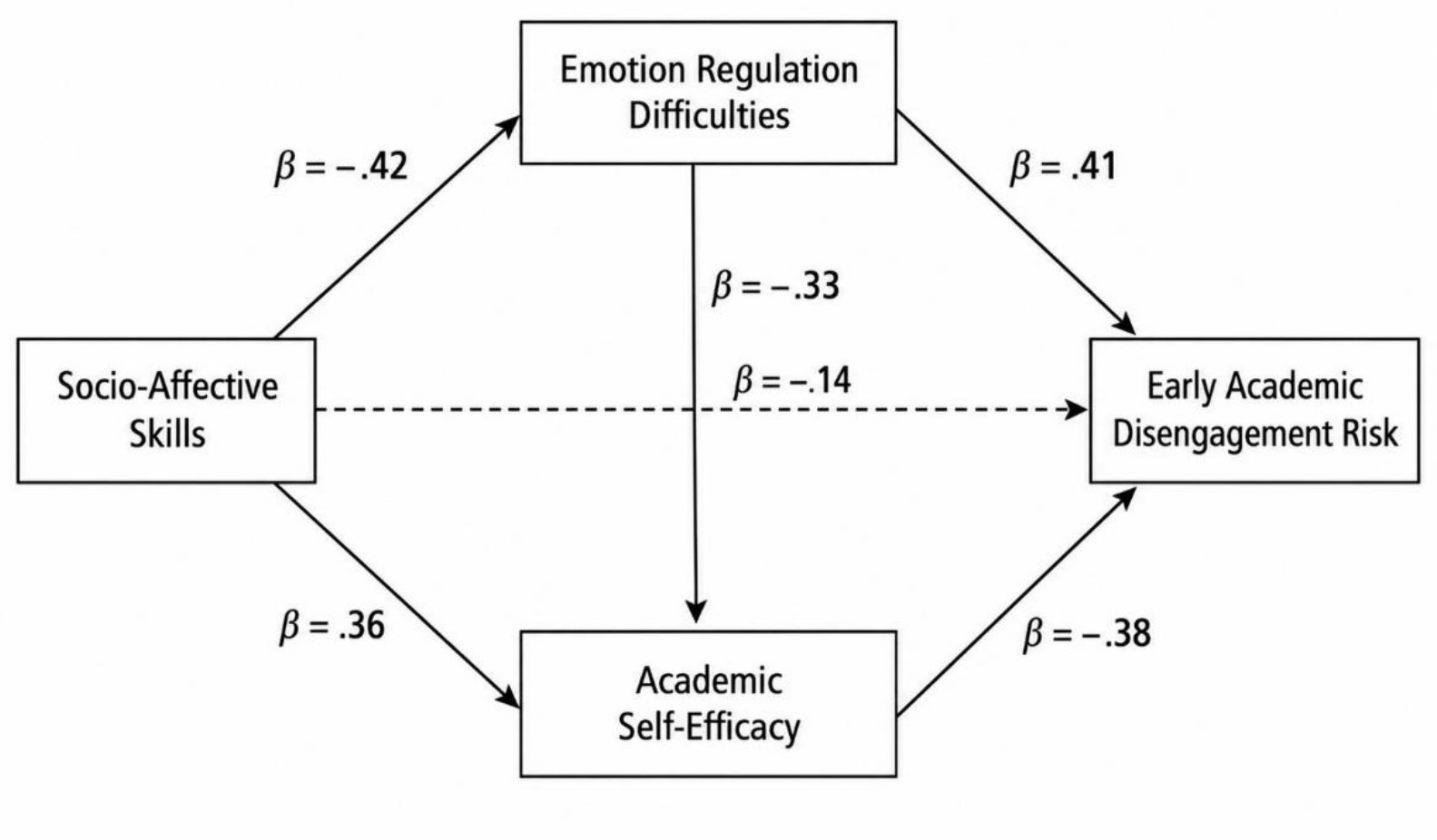
Structural equation model illustrating the hypothesized associations among socio-affective skills, emotion regulation difficulties, academic self-efficacy, and early academic disengagement risk. Values represent standardized path coefficients (β).

The hypothesized structural model demonstrated good fit to the data, χ^2^ (231) = 548.32, *p* < 0.001, *CFI* = 0.95, *TLI* = 0.94, *RMSEA* = 0.038 (90% *CI* [0.034, 0.042]), *SRMR* = 0.041. Socio-affective skills were negatively associated with emotion regulation difficulties (β = −0.42, *p* < 0.001) and positively associated with academic self-efficacy (β = 0.36, *p* < 0.001). Emotion regulation difficulties were negatively associated with academic self-efficacy (β = −0.33, *p* < 0.001) and positively associated with early academic disengagement risk (β = 0.41, *p* < 0.001). Academic self-efficacy was negatively associated with early academic disengagement risk (β = −0.38, *p* < 0.001). The direct effect of socio-affective skills on early academic disengagement risk remained significant after accounting for the mediators (β = −0.14, *p* = 0.004), indicating partial mediation. The hypothesized relationships among the study variables are presented in Figure 1. The standardized direct effects estimated in the structural model are presented in Table 2.

**Table 2:** Standardized direct effects in the structural model.

Path	β	*SE*	*p*
Socio-affective skills → emotion regulation difficulties	−0.42	0.04	< 0.001
Socio-affective skills → academic self-efficacy	0.36	0.05	< 0.001
Emotion regulation difficulties → academic self-efficacy	−0.33	0.04	< 0.001
Emotion regulation difficulties → early academic disengagement risk	0.41	0.05	< 0.001
Academic self-efficacy → early academic disengagement risk	−0.38	0.05	< 0.001
Socio-affective skills → early academic disengagement risk	−0.14	0.05	0.004

### Mediation analyses

Bootstrap analyses indicated statistically significant indirect associations linking socio-affective skills with early academic disengagement risk through academic self-efficacy (indirect effect = −0.14, 95% *CI* [−0.19, −0.10]) and emotion regulation difficulties (indirect effect = −0.17, 95% *CI* [−0.22, −0.12]).

Importantly, a significant sequential indirect effect was observed, supporting the proposed mediation pathway from socio-affective skills to early academic disengagement risk through emotion regulation difficulties and academic self-efficacy (indirect effect = −0.05, 95% *CI* [−0.08, −0.03]). Because the confidence interval did not include zero, the sequential mediation hypothesis was supported. The indirect effects obtained from the mediation analyses are summarized in Table 3.

**Table 3:** Indirect effects of socio-affective skills on early academic disengagement risk.

Indirect pathway	Effect	95% *CI*
Via emotion regulation difficulties	−0.17	[−0.22, −0.12]
Via academic self-efficacy	−0.14	[−0.19, −0.10]
Via emotion regulation → academic self-efficacy	−0.05	[−0.08, −0.03]

### Summary of hypothesis testing

All proposed hypotheses were supported at the level of observed associations and indirect effects. Socio-affective skills were associated with lower early academic disengagement risk both directly and indirectly through emotion regulation and academic self-efficacy. The findings are consistent with a sequential mediation model in which socio-affective skills function as distal protective resources associated with lower disengagement risk through emotional regulation and academic self-efficacy processes.

## Discussion

The present study examined the psychological pathways linking socio-affective skills to early academic disengagement risk through emotion regulation and academic self-efficacy. The findings are broadly consistent with the proposed sequential mediation framework and align with a growing body of research conceptualizing disengagement as a gradual psychological process. Given the cross-sectional nature of the design, however, the results should be interpreted as theoretically consistent associations rather than confirmation of temporal or causal pathways. By empirically integrating socio-affective skills with emotion regulation and academic self-efficacy, this study extends contemporary disengagement models and offers novel insights into early dropout risk in higher education.

Consistent with previous studies, socio-affective skills were negatively associated with early academic disengagement risk, reinforcing evidence that interpersonal and emotional competencies serve as protective resources during the transition to university. Recent research has demonstrated that students with stronger socio-affective competencies report higher academic engagement and lower disengagement, particularly in the first year of study (Al-Maamari et al., 2025; Taylor et al., 2017). Importantly, the partial mediation observed in the present model suggests that socio-affective skills were not only directly associated with disengagement risk but also indirectly associated with it through self-regulatory variables, a finding that aligns with recent theoretical advances in self-regulation and student engagement research (Putwain et al., 2022).

Importantly, early academic disengagement risk is conceptualized here as a psychological disengagement process rather than a proxy for dropout intention, emphasizing its role as an early, malleable target for prevention.

Emotion regulation emerged as a central mediator in the relationship between socio-affective skills and early academic disengagement risk. This finding is consistent with recent empirical evidence indicating that difficulties in regulating academic emotions—such as anxiety, frustration, and hopelessness—are strongly associated with academic disengagement, avoidance behaviors, and other maladaptive academic behaviors (Harley et al., 2019), which have been linked to dropout intentions in longitudinal research (Zhou and Ee, 2012; Álvarez-Pérez et al., 2024). Moreover, longitudinal studies have shown that emotional dysregulation predicts declines in engagement over time, even after controlling for prior academic performance (Álvarez-Pérez et al., 2024). By situating emotion regulation within a mediation framework, the present study extends this literature by suggesting its potential role as a key psychological mechanism translating socio-affective resources into reduced disengagement risk.

The positive association between socio-affective skills and emotion regulation capacity observed in this study is also supported by recent research. For example, Liu et al. (2022) found that social–emotional competence was positively associated with adaptive emotion regulation strategies and negatively associated with academic stress among university students. Similarly, Al-Maamari et al. (2025) emphasized that socio-affective skills enhance students' ability to identify, interpret, and manage emotional responses in socially and academically demanding contexts. Together, these findings support the interpretation that socio-affective skills facilitate more effective emotional coping, which may be associated with lower vulnerability to early academic disengagement.

Academic self-efficacy also played a significant mediating role, corroborating extensive recent evidence identifying self-efficacy as a critical determinant of academic persistence and engagement. Systematic reviews and meta-analyses have consistently shown that students with higher academic self-efficacy are less likely to disengage, procrastinate, or consider dropout, even when facing academic challenges (Honicke and Broadbent, 2023). In line with this literature, the present findings indicate that higher self-efficacy is associated with lower early academic disengagement risk, highlighting the importance of motivational beliefs in sustaining academic involvement.

Notably, the results further revealed a strong negative association between emotion regulation difficulties and academic self-efficacy. This pattern is consistent with control–value theory, which argues that persistent difficulties in regulating achievement emotions undermine perceptions of control and confidence, thereby weakening academic self-efficacy and increasing disengagement risk (Liu et al., 2022; Pekrun, 2021). For instance, (Liu et al. 2022) showed that emotion regulation predicted subsequent changes in academic self-efficacy, which in turn influenced persistence-related outcomes. The present study extends this work by positioning emotion regulation as a precursor to self-efficacy within a broader disengagement pathway.

The significant sequential indirect association observed in this study was consistent with the proposed theoretical framework linking socio-affective skills, emotion regulation difficulties, academic self-efficacy, and early academic disengagement risk. However, because temporal ordering cannot be established using cross-sectional data, the findings should be interpreted as supporting a theoretically plausible pattern of associations rather than a confirmed developmental process.

These findings are consistent with recent calls for process-oriented models that capture the dynamic interplay between emotional and motivational factors in higher education (Pedler et al., 2022; Rump et al., 2023; Gilani and Thomas, 2025). Rather than treating emotion regulation and self-efficacy as isolated predictors, the present findings suggest that these mechanisms may operate jointly in patterns associated with early academic disengagement.

From a theoretical standpoint, these results contribute to the literature in several ways. First, they advance the conceptualization of early academic disengagement (previously referred to as silent dropout processes) by empirically linking it to well-established psychological mechanisms, thereby supporting its psychological plausibility as an early disengagement risk indicator, while underscoring the need for longitudinal and predictive validation in future research against subsequent dropout intentions and administrative outcomes.

Second, they integrate social–emotional learning frameworks with self-regulation and social cognitive theory, responding to recent calls for interdisciplinary models of student engagement and persistence (Al-Maamari et al., 2025). Third, the sequential mediation model offers a refined understanding of how distal socio-affective resources translate into proximal motivational and emotional processes that protect against disengagement.

Although academic field did not emerge as a significant predictor of early academic disengagement risk in the final model, disciplinary differences remain theoretically relevant. Students enrolled in different academic fields may experience distinct learning environments, workload demands, evaluation practices, and professional socialization processes that shape their academic experiences. For example, highly structured programs such as Health Sciences may involve intensive evaluation and performance pressures, whereas creative disciplines may present different motivational and self-regulatory challenges. These possibilities remain speculative and warrant empirical examination in future research. Future research should examine whether the psychological pathways identified in the present study operate similarly across academic disciplines and whether specific disciplinary contexts amplify or attenuate disengagement risk.

The findings also have important practical implications. Universities could implement brief screening protocols during the first academic year to identify students experiencing elevated emotion regulation difficulties and low academic self-efficacy, both of which were associated with greater disengagement risk in the present study. Targeted psychoeducational interventions focused on emotional regulation, coping skills, and stress-management strategies could be incorporated into first-year support programs. Additionally, academic advisors and student-support personnel could receive training to recognize early indicators of psychological disengagement, such as reduced participation, avoidance of evaluative situations, and declining academic confidence. Peer-mentoring initiatives may also provide valuable socio-emotional resources that facilitate adaptation to university demands and reduce vulnerability to early academic disengagement.

In conclusion, the present study provides evidence that socio-affective skills are associated with lower early academic disengagement risk through interconnected emotional and motivational variables. By linking silent dropout processes with theoretically grounded psychological mechanisms, this research contributes to theory-driven approaches for early identification and prevention of disengagement in higher education, emphasizing the central roles of emotion regulation and academic self-efficacy in sustaining student persistence.

From an educational perspective, these findings suggest that socio-affective learning and emotional regulation training may represent promising targets for university support programs, although intervention and longitudinal research is required before drawing conclusions regarding effectiveness. Future intervention research should examine whether strengthening these competencies contributes to improvements in academic functioning, engagement, and persistence. Such studies may help determine the potential value of socio-affective competencies as targets for evidence-based educational interventions aimed at promoting student engagement.

## Limitations and future research

Despite the strengths of this study, several limitations should be acknowledged. First, the cross-sectional design restricts the ability to draw causal inferences regarding the directionality of the observed relationships. Although the proposed sequential mediation model is theoretically grounded and consistent with prior longitudinal evidence, it is not possible to determine temporal precedence among socio-affective skills, emotion regulation, academic self-efficacy, and early academic disengagement risk. Future research should employ longitudinal designs to examine how these psychological processes evolve over time and to assess whether changes in socio-affective skills and emotion regulation predict subsequent trajectories of disengagement and actual dropout behavior.

Second, the reliance on self-report measures may introduce common method variance and social desirability bias. While all instruments used in the present study demonstrated adequate reliability and have been widely validated in higher education contexts, future studies would benefit from incorporating multi-method approaches. For instance, combining self-report data with behavioral indicators (e.g., learning management system activity, attendance patterns) or institutional records could provide a more comprehensive assessment of silent dropout processes and reduce shared method bias.

Third, the operationalization of early academic disengagement risk, although grounded in recent process-oriented models of disengagement, remains an emerging construct. While the present findings support its psychological validity, further research is needed to refine its measurement and to examine its predictive utility for long-term academic outcomes, including formal dropout, academic delay, and re-enrollment. Longitudinal validation studies would be particularly valuable in establishing early academic disengagement risk as a reliable early warning indicator within higher education systems.

Fourth, the sample was drawn from a single large private university in Mexico, which may limit the generalizability of the findings. Institutional characteristics, such as admission policies, academic support structures, and student demographics, may influence the manifestation of disengagement processes. Future research should replicate the proposed model across diverse institutional contexts, including public universities and institutions in other cultural settings, to examine the robustness and cross-cultural applicability of the identified psychological pathways.

Furthermore, although academic field was included as a control variable, the present study was not specifically designed to examine disciplinary differences. Future research should investigate whether the proposed psychological pathways operate differently across academic disciplines characterized by distinct curricular structures, learning environments, and performance demands.

Finally, although the present study focused on socio-affective skills, emotion regulation, and academic self-efficacy as central mechanisms, disengagement and dropout are inherently multifactorial phenomena. Future studies should consider integrating contextual and structural variables—such as teaching practices, perceived institutional support, financial stress, or family responsibilities—into more comprehensive models. Additionally, examining potential moderators (e.g., academic field, gender, first-generation student status) may help identify subgroups for whom these psychological pathways are particularly salient.

In terms of future directions, intervention-based research represents a critical next step. Experimental or quasi-experimental studies evaluating programs designed to strengthen socio-affective skills and emotion regulation during the first year of university could provide direct evidence of the causal role of these competencies in reducing early academic disengagement risk. Such research would not only advance theoretical understanding but also help determine whether these competencies represent viable targets for evidence-based retention strategies in higher education.

## Data Availability

The raw data supporting the conclusions of this article will be made available by the authors, without undue reservation.
