# Supplementary material for: Socio-affective skills and early academic disengagement in higher education: a sequential mediation model of emotion regulation and academic self-efficacy
Source: Front Psychol. 2026 Jul 23;17:1849945. doi: 10.3389/fpsyg.2026.1849945 (PMC13444892; doi:10.3389/fpsyg.2026.1849945)
Supplement: Supplementary file 1 [file Supplementary_File_1.docx]

**SUPPLEMENTARY MATERIALS**

**Supplementary Table S1**

**Reliability Estimates for SECQ and DERS Dimensions**

| Instrument | Dimension | McDonald's ω |
| --- | --- | --- |
| SECQ | Self-awareness | .84 |
| SECQ | Social awareness | .82 |
| SECQ | Self-management | .86 |
| SECQ | Relationship management | .85 |
| SECQ | Responsible decision-making | .81 |
| DERS | Nonacceptance | .87 |
| DERS | Goals | .84 |
| DERS | Impulse | .85 |
| DERS | Awareness | .76 |
| DERS | Strategies | .89 |
| DERS | Clarity | .81 |

*Note*. Reliability estimates are reported using McDonald's omega (ω) and were calculated using the final analytic sample (N = 942).

**Supplementary Table S2**

**Academic Self-Efficacy Parcel Allocation**

| Parcel | Items Included |
| --- | --- |
| Parcel 1 | ASE1, ASE4 |
| Parcel 2 | ASE2, ASE5 |
| Parcel 3 | ASE3, ASE6 |

*Note*. Parcels were created using the item-to-construct balance approach (Little et al., 2002).

**Supplementary Table S3**

**Early Academic Disengagement Risk Parcel Allocation**

| Parcel | Items Included |
| --- | --- |
| Parcel 1 | EADR1, EADR4, EADR7 |
| Parcel 2 | EADR2, EADR5, EADR8 |
| Parcel 3 | EADR3, EADR6 |

*Note*. Parcel construction followed the item-to-construct balance procedure recommended for SEM applications involving unidimensional latent variables.

**Supplementary Appendix A**

**Early Academic Disengagement Risk Scale (EADRS)**

Response Scale

1 = Strongly disagree
2 = Disagree
3 = Neither agree nor disagree
4 = Agree
5 = Strongly agree

EADR1. I often feel emotionally disconnected from my academic activities.

EADR2. I sometimes question whether continuing my studies is worthwhile.

EADR3. I find it increasingly difficult to remain engaged with coursework.

EADR4. I frequently postpone academic responsibilities even when they are important.

EADR5. I feel less motivated to invest effort in my studies than I did when I started university.

EADR6. I avoid seeking academic support even when I need help.

EADR7. I feel less connected to my university community than I would like.

EADR8. I occasionally think about leaving university before completing my degree.

**Supplementary Appendix B**

**Scoring Procedure for the Early Academic Disengagement Risk Scale**

The total EADRS score is calculated by averaging responses across the eight items. Higher scores indicate greater vulnerability to early academic disengagement. No reverse-scored items were included. For structural equation modeling analyses, parcels were constructed according to the allocation procedure reported in Supplementary Table S3. The EADRS was conceptualized as an indicator of early psychological disengagement while students remain formally enrolled.
